# Carvacrol attenuated lipopolysaccharide-induced intestinal injury by down-regulating TLRs gene expression and regulating the gut microbiota in rabbit

**DOI:** 10.1038/s41598-023-38577-w

**Published:** 2023-07-15

**Authors:** Diange Wu, Miao Xia, An Yan, Haotian Jiang, Jiaqi Fan, Siyuan Zhou, Xu Wei, Shudong Liu, Baojiang Chen

**Affiliations:** College of Animal Science and Technology, Hebei Agricultural University, No 2596, Lekai South Street Nanshi District, Baoding, 071000 China

**Keywords:** Immunology, Inflammation, Acute inflammation

## Abstract

Carvacrol (CAR) is a plant extract that has been reported to enhance antioxidant activity in animals. However, the effect of CAR on the intestinal health of rabbits is poorly understood. Here, we investigated whether CAR exerts protective effects on the intestinal health of rabbits following lipopolysaccharide (LPS) challenge and whether these effects were mediated via the reduction of intestinal inflammation and the regulation of the intestinal flora. Intestinal damage was assessed in LPS-challenged rabbits treated or not with CAR. The serum levels of inflammatory factors were assessed by enzyme-linked immunosorbent assay. Histopathological changes in the ileum and cecum were examined using hematoxylin and eosin staining. The relative gene expression levels of inflammatory factors and tight junction proteins in the rabbit cecum were determined by qRT-PCR. High-throughput sequencing analysis of the microbial 16S rRNA gene was performed using the Illumina NovaSeq Platform. The results showed that CAR can prevent intestinal inflammation and damage as well as mitigate gut dysbiosis in rabbits following LPS challenge. Our study provides a theoretical reference for the application of dietary CAR in rabbit production.

## Introduction

The intestinal tract is the main organ responsible for nutrient digestion and absorption as well as the first line of defense against pathogenic microorganisms and toxins^1^. This line of defense encompasses both mechanical and biological barriers. The mechanical barrier comprises intestinal epithelial cells, which separate the intestinal lumen from intestinal tissue, and tight junctions (TJs), consisting of proteins such as zonula occludens-1 (ZO-1), occludin, and claudin. In addition to organizing epithelial cells into apical and basal compartments and maintaining epithelial cell polarity, TJ proteins also regulate the permeability of cells to ions, water, and nutrients, thus preserving intestinal homeostasis^2–4^. The biological barrier, meanwhile, comprises a micro-ecosystem consisting of resident bacteria that are interdependent and exist in a symbiotic relationship with their host. Following pathological insult, however, this biological barrier can be disrupted, resulting in the translocation of bacteria and endotoxins from the intestinal lumen to intestinal tissue or the circulation, consequently leading to infection or intestinal inflammation^5^. During the breeding process, animals are subjected to a variety of stresses, such as uncomfortable living environments, differences in feed, and transportation, which affect the structure of their intestinal flora^6–8^. This dysbiosis is mainly manifested as an increase in the abundance of harmful microorganisms in the intestine and the production and release of large amounts of lipopolysaccharide (LPS), which is detrimental to health^9^.

LPS is a constituent of the outer wall of Gram-negative bacteria^10^ and comprises an O-antigen, a core oligosaccharide, and a lipid anchor (lipid A)^11^. Lipid A is the most conserved region of LPS. It is made up of six saturated acyl chains, which are double phosphorylated and are responsible for the proinflammatory effects of LPS. Furthermore, the abundant anion groups present in the core region of lipid A combine with divalent cations (Mg^2+^ and Ca^2+^), which increases lipid A molecular density, improves bacterial outer membrane stability, reduces permeability, and effectively resists the destructive effects of bacteriophages or bacteriocins^12^. In the body, LPS is recognized by LPS-binding protein (LBP), and is subsequently transported to the surface of immune cells, where it activates the Toll-like receptor 4 (TLR4) signaling pathway, leading to the production of cytokines and, consequently, an inflammatory response^13^. In vitro, 1 μg/mL LPS was shown to significantly downregulate the expression of occludin and claudin 1 in porcine intestinal epithelial cells (IPEC-J2)^14^, while in Caco-2 cells, treatment with 10 μg/mL LPS for 24 h significantly reduced the mRNA levels of *ZO-1*, claudin 1, and occludin, and activated the proinflammatory TLR4/NF-κB signaling pathway^15^. In vivo, intramuscular injection of LPS (500 μL/kg body weight) in broilers significantly increased the mRNA expression of interleukin 1 beta (*IL-1β*), *IL-6*, *IL-8*, and tumor necrosis factor alpha (*TNF-α*) in the small intestine, and activated the TLR/NF-κB pathway 2 h post-injection^16^.

Antibiotics act by killing bacteria or inhibiting their growth, thereby reducing the secretion of harmful substances produced by these bacteria, such as LPS, and mitigating their negative effects. Accordingly, antibiotics have long been used to inhibit bacterial proliferation and treat bacterial infections. However, antibiotics and their residues are associated with side effects, such as resistance and variability of effects and the destruction of animal intestinal microecology^17,18^. This highlights the need for the development of alternatives to antibiotics in the animal industry to alleviate the impact of harmful bacteria^19^. Among the many alternatives, plants and their constituents have become research hotspots owing to their antibacterial effects^20^, as well as their ability to alleviate inflammatory responses^21^ and improve intestinal health^22^.

The use of plant-based feed additives to alleviate or treat intestinal inflammation or intestinal injury caused by LPS has been widely studied in several species but not in rabbits. Carvacrol (CAR) is a monophenol widely present in various plants, fruits, and volatile oils, including oregano, thyme, and olive fruit^23^. Studies have shown that CAR has significant anticancer^24^ and antioxidative properties^25^; however, it is unclear whether CAR supplementation can protect the intestine of rabbits from LPS challenge. In this study, we hypothesized that dietary CAR can alleviate intestinal injury induced by LPS in rabbits by alleviating intestinal inflammation and regulating intestinal microflora.

## Materials and methods

### Experimental animals, diet, and design

All experiments were performed in accordance with the ARRIVE guidelines and were carried out following the U.K. Animals (Scientific Procedures) Act, 1986 and associated guidelines; EU Directive 2010/63/EU for animal experiments; and the National Institutes of Health Guide for the Care and Use of Laboratory Animals (NIH Publications No. 8023, revised 1978) and were approved by the Ethics Committee of Animal Experimentation of Hebei Agricultural University (Protocol 2020006).

A total of 40-day-old Ira rabbits (20 male, 20 female; purchased from China Hebei Xingtai Weixian Kangming culture Co., LTD) of similar weight (1.25 kg) were placed in battery cages, one rabbit per cage. During the experiment, the temperature and photoperiod were maintained according to commercial conditions (20–23 °C and 12-h:12-h light/dark cycle, respectively). Each cage was equipped with one feeder and one nipple drinker. Each treatment group contained ten replicates (cages), with one sampling unit (rabbit) per cage. The basal diet was formulated to meet the nutrient and energy requirements of rabbits according to De Blas and Wiseman^26^. The CAR diet was formulated by adding 25 g/t CAR to the basal diet. CAR (purity ≥ 98%) was purchased from Fidi Feed Technology Co., Ltd (Beijing, China). All the diets were pelleted to a size of 4 × 10 mm (diameter × length). The composition and nutrient levels of the basal diet are shown in Table 1. The trial lasted for 28 days.

**Table 1:** Composition and nutrient levels of the basal diet (dry matter) %

Ingredients	Content	Nutritional level^b^	Content
Corn	8.00	DE/(MJ/kg)	10.35
Wheat bran	33.00	CP	15.42
Rice bran	5.00	CF	20.09
Soybean meal	11.00	EE	2.96
Peanut vine	15.00	Ca	1.43
Peanut shell	12.50	TP	0.58
Artemisia argyi powder	8.00	NDF	40.24
Wheat middlings	3.50	ADF	22.77
Premix^a^	4.00	Met	0.20
Total	100.00	Lys	0.31
		ADL	6.03

^a^The premix provided the following per kg of diets: Cu (as copper sulfate) 20 mg, Fe (as ferric sulfate) 70 mg, Zn (as zinc sulfate) 70 mg, Se (as selenium sulfate) 0.25 mg, Mn (as manganese sulfate) 10 mg, Co 0.15 mg, I 0.2 mg, VB1 2 mg, VB2 6 mg, VB12 0.02 mg, Pyridoxine 2 mg, Pantothenic acid 50 mg, Nicotinic acid 50 mg, Choline 1000 mg, Biotin 0.2 mg, VA 10 000 IU, VD 900 IU, VE 50 mg, VK 2 mg.

^b^DE was a calculated value, while the others were measured values.

A 2 (with or without CAR) × 2 (with or without LPS) factorial arrangement of treatments in a completely randomized design was used in this study. Individually housed rabbits were randomly assigned to four treatment groups, as follows: A CON group (basal diet + saline), a CAR group (basal diet + carvacrol + saline), a LPS group (basal diet + LPS), and a CAR + LPS group (basal diet + CAR + LPS).

On day 28 of the trial (68 days of age), the rabbits assigned to the LPS groups were intramuscularly (thigh muscle) injected with 1 mL (200 μg/kg body weight) of *Escherichia coli* LPS (*Escherichia coli* O55: B5, L2880, Sigma-Aldrich). The remaining rabbits were injected with 1 mL of 0.9% saline (all injections were performed simultaneously at 08:00 h and all the animals were euthanized and sampled simultaneously at 12:00 h).

### Sample collection

All the rabbits from each group were sampled 4 h post-injection. Blood samples were drawn from the ear vein using a sterile blood-sampling needle and centrifuged at 3500×*g* for 10 min at 4 °C to obtain serum. Serum samples were then immediately stored at − 80 °C until further analysis. Sodium pentobarbital was used as an anesthetic and was administered intravenously. The ileal and cecal segments were collected, washed, and immediately immersed in a 4% paraformaldehyde solution for histological examination. The cecum and cecal chyme were stored at − 80 °C for further analysis.

### Measurement of inflammatory indicators in serum

Serum IL-1β, IL-6, IL-8, and TNF-α levels were detected using the respective enzyme-linked immunosorbent assay (ELISA) kits (Jiangsu Meimian Industrial Co., Ltd, Jiangsu, China).

### Structural morphology of the intestine

The ileal and cecal segments were fixed in a 4% paraformaldehyde solution for 24 h, dehydrated, embedded in paraffin, cut into 5-μm-thick sections using a microtome (Kedee KD2508, Kedee BInstruments and Equipment Co., Ltd, Jinhua, China), fixed on slides, and finally stained with hematoxylin and eosin. Images of the ileum and cecum were acquired with a Nikon Eclipse E100 microscope (Nikon Inc., Tokyo, Japan) and analyzed with CaseViewer software (version 2.4, ServiceBio, Wuhan, China). Five sections of the slice were randomly selected from each sample for assessment of morphology. The average of five values from each rabbit was used for statistical analysis.

### Quantitative reverse transcription-polymerase chain reaction (qRT-PCR)

The mRNA expression of *ZO-1*, *occludin*, *claudin-1*, *IL-1β*, *IL-6*, *IL-8*, *TNF-α*, Toll-like receptor 2 (*TLR2*), *TLR4*, *myeloid differentiation primary response 88* (*MYD88*), *nuclear factor kappa B* (*NF-κB*), mitogen-activated protein kinase 1 (*MAPK1*), *MAPK8*, and *MAPK14* was detected by qRT-PCR. The primers used in this study are listed in Table 2. The mRNA sequences were obtained from the National Center for Biotechnology Information (NCBI) (http://www.ncbi.nlm.nih.gov/cgi-bin/bank). Total RNA was extracted from the cecum using Trizol as described by the manufacturer (TaKaRa, Dalian, China)^27^. Total RNA concentration and purity were determined by measuring the optical density at 260 and 280 nm. The extracted mRNA was reverse transcribed into cDNA according to the manufacturer's instructions (TaKaRa, Dalian, China). The reverse transcription conditions were 42 °C for 40 min and 65 °C for 10 min. Quantitative PCR involved a pre-denaturation step at 95 °C for 5 min, followed by 40 cycles of 95 °C for 10 s and 60 °C for 30 s. A melt curve analysis was applied at the end of the run to determine specific product amplification. Relative gene expression levels were evaluated using the 2^−ΔΔCT^ method^16^ (CAR-treated samples *versus* the non-supplemented [control] samples) after normalization to the housekeeping gene.

**Table 2:** Primer information.

Gene	Accession no.	Forward primer (5′–3′)	Reverse primer (5′–3′)
*ZO-1*	100346390	GAGAACAAGAAGGAGGTGAA	CACTGAACTGGCTCTGAG
*Occluding*	100338492	TTGAGCAGCAGCAGTAAC	TGTAGTCCGTCTCGTAGTG
*Claudin-1*	NM_001089316	AATTCGGTCAGGCTCTTT	GAGGACAAGAACAGCAAAG
*NF-κB*	XM-008272930	GGCTCAACATCTACACAGT	GTCCTTGCGGAAGTCAAT
*IL-1β*	NM_001082201	GAATTTGAGTCTGCCCAGTT	CCATGCTGAAGTCAATTAGGT
*IL-6*	NM_001082064	CAAGTTCAGGAGTGACGAA	GAGGGTGGCTTCTTCATT
*IL-8*	NM_001082293	TGATGGAAGAGAACTCTGC	ATGACTCTTGCTGCTCAG
*TNF-α*	NM_001082263	AAGGTCAACCTCCTCTCT	CAGATAGATGGGCTCATACC
*TLR2*	NM_001082781.1	AGTACCAGAGGGTGTTACTG	GTAGCTCTTAGCACCTGTTC
*TLR4*	NM_001082732.2	AGATTGCTCAGACCTGGCAG	CACTGAGTCCAGAGGGAATG
*MYD88*	NW_003159756.1	GACTCCTGAGTGCCTCAGC	CTGCAGTCCCAGGGACCAG
*MAPK14*	XM_017345066.1	TGACCTGCTGGAGAAGATGC	CTGCTCATAGACGAGTGGAA
*MAPK8*	XM_002722670.3	GAGTGTGTTCTTACTTCATG	CCAAGTGTTGTGACTGTATC
*MAPK1*	XM_017349593.1	CCCTTCACAAGAAGACCTGA	CGAAGCTCCATTCAAGTTCG
*GAPDH*	NM_001082253.1	CTGAACGGGAAACTCACT	TCACCACCTTCTTGATGTC

*ZO-1*: zonula occludens-1; *IL-1β*: interleukin 1 beta; *IL-6*: interleukin 6; *IL-8*: interleukin 8 beta; *TNF-α*: tumor necrosis factor alpha; *TLR2*: toll-like receptor 2; *TLR4*: toll-like receptor 4; *MYD88*: myeloiddifferentiationfactor 88; *NF-κB*: nuclear factor kappa-B; *MAPK8*: mitogen-activated protein kinase 8; *MAPK14*: mitogen-activated protein kinase 14; *MAPK1*: mitogen-activated protein kinase 1.

### Short-chain fatty acid (SCFA) analysis

Cecal digesta (0.5 g) was diluted (dilution factor of 3) and centrifuged at 12,000 rpm for 10 min at 4 °C. Then, 1 mL of supernatant was mixed with 0.2 mL of 25% ammonium metaphosphate solution containing the internal standard 2-ethylbutyric acid (2 EB), the mixture was soaked in ice for 40 min and then centrifuged at 12,000 rpm for 10 min at 4 °C to remove protein precipitates. The supernatant was used for SCFA analysis using a gas mass spectrometer (GC2014, Shimadzu Inc., Tokyo Japan).

### 16S rRNA gene sequencing and analysis

Total genomic DNA was extracted from cecal digesta using the OMEGA Soil DNA Kit (M5635-02; Omega Bio-Tek, Norcross, GA, USA) following the manufacturer’s instructions and was stored at − 20 °C before analysis. The quantity and quality of the extracted DNA were assessed using a NanoDrop NC2000 spectrophotometer (Thermo Fisher Scientific, Waltham, MA, USA) and agarose gel electrophoresis, respectively. PCR amplification of the V3–V4 region of the bacterial 16S rRNA gene was performed using forward primer 338F (5′-ACTCCTACGGGAGGCAGCA-3′) and reverse primer 806R (5′-GGACTACHVGGGTWTCTAAT-3′). The reverse primer contained a sample-specific 7-nucleotide barcode. Amplification was performed using the following program: 98 °C for 5 min, followed by 25 cycles of 98 °C for 30 s, 53 °C for 30 s, and 72 °C for 45 s, with a final extension at 72 °C for 5 min. The amplicons were purified with Vazyme VAHTSTM DNA Clean Beads (Vazyme, Nanjing, China) and quantified using the Quant-iT PicoGreen dsDNA Assay Kit (Invitrogen, Carlsbad, CA, USA). After individual quantification, the amplicons were pooled in equal amounts, and paired-end sequenced (2 × 250 bp) at Shanghai Personal Biotechnology Co., Ltd (Shanghai, China) on the Illumina NovaSeq Platform using the NovaSeq 6000 SP Reagent Kit (500 cycles).

Analysis of sequencing data was mainly performed using QIIME2 and R software (v3.2.0). To estimate microbial diversity in individual samples, alpha diversity indices, such as the Chao1 richness estimator, Shannon diversity index, Simpson index, and Faith’s PD, at the amplicon sequence variant (ASV) level were calculated using the ASV table in QIIME2. Principal coordinate analysis (PCA) was performed based on Bray–Curtis and UniFrac distance metrics.

### Data analysis

All data were analyzed using Statistical Package for the Social Sciences (SPSS) ver. 21 and were expressed as means ± SEM. Differences among groups were examined using one-way analysis of variance (ANOVA) followed by Duncan’s multiple-range test. Differences were considered significant at *P* < 0.05.

## Results

### Levels of inflammatory cytokines in serum

**Figure 1:**
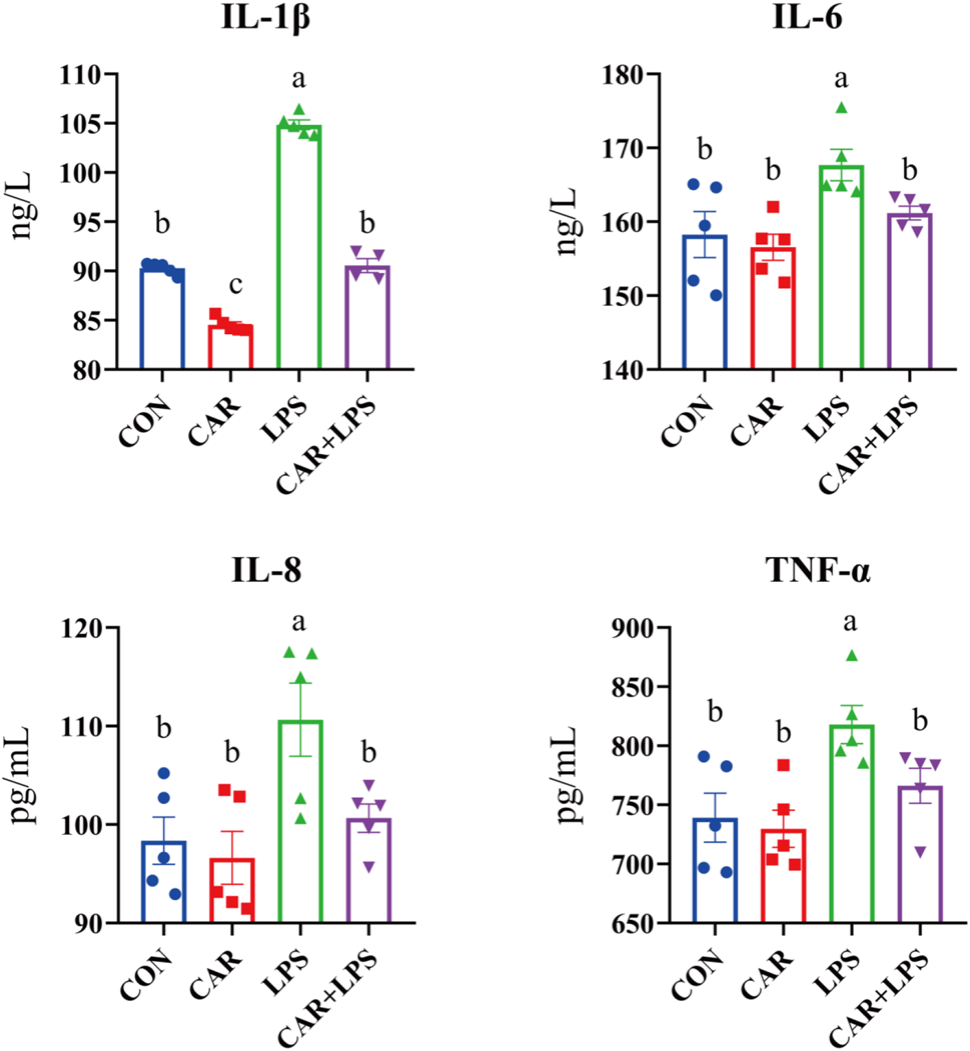
Effects of CAR on the levels of inflammatory cytokines in the serum of LPS-challenged rabbits. Values are the means of five replicates per treatment.

As shown in Fig. 1, LPS challenge increased the levels of IL-1β, IL-6, IL-8, and TNF-α in serum (*P* < 0.05), whereas the addition of CAR to the diet reduced the serum levels of IL-1β (*P* < 0.05). Additionally, dietary CAR alleviated the increase in IL-1β, IL-6, IL-8, and TNF-α levels resulting from LPS challenge (*P* < 0.05).

### Expression of inflammatory genes in the cecum

**Figure 2:**
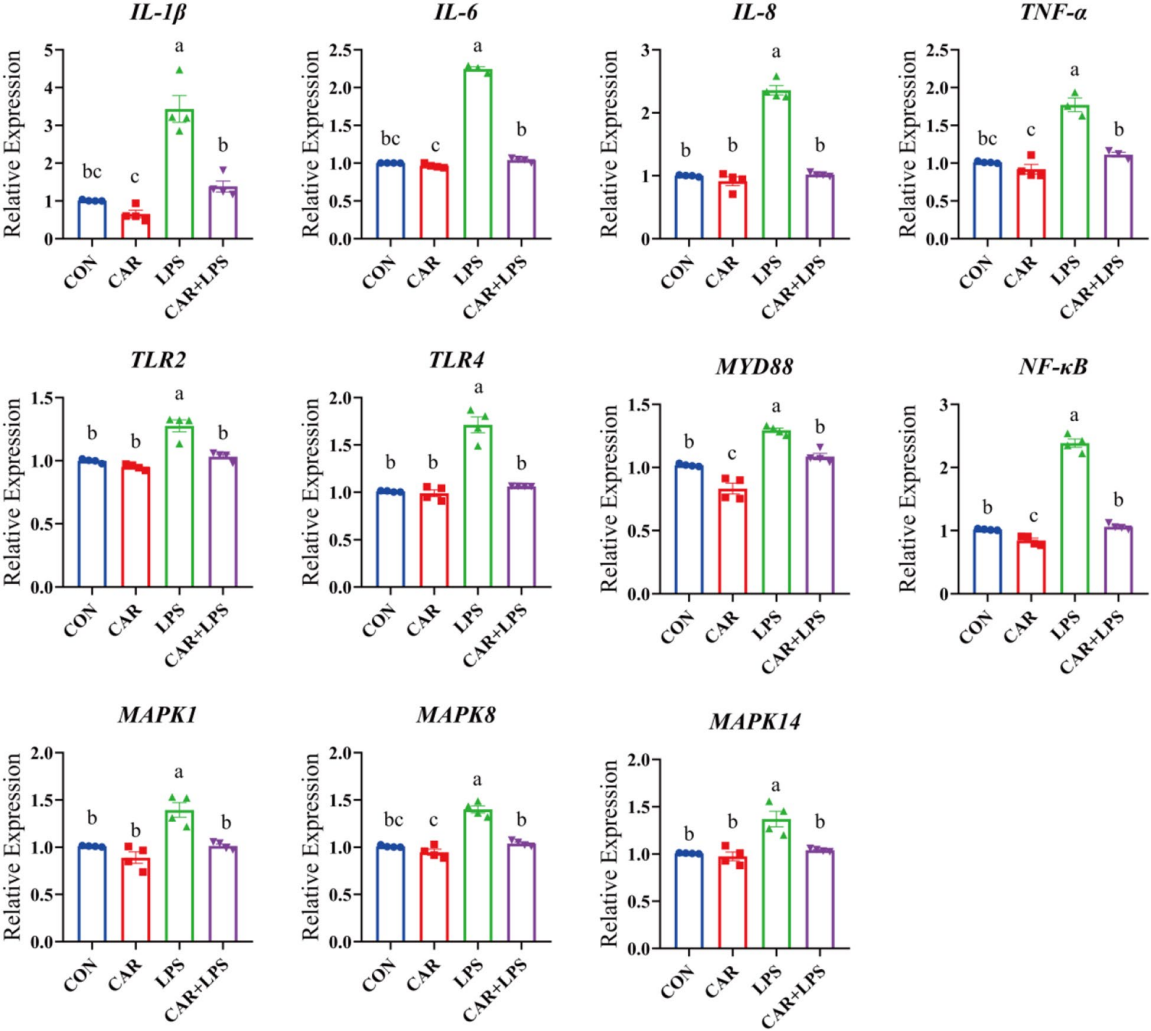
Effects of CAR on the expression of inflammatory factors gene in the cecum of LPS-challenged rabbits. ^a,b,c^Within a row, means with no common superscripts differ significantly (*P* < 0.05). Values are the means of four replicates per treatment.

To determine the genetic basis of the CAR-mediated improvement of the inflammatory reaction in LPS-challenged rabbits, the expression of proinflammatory genes and genes involved in the inflammatory signaling pathway was investigated by qPCR (Fig. 2). LPS treatment upregulated the expression of the *IL-1β*, *IL-6*, *IL-8*, *TNF-α*, *TLR2*, *TLR4*, *MYD88*, *NF-κB*, *MAPK1*, *MAPK8*, and *MAPK14* genes (*P* < 0.05). In contrast, dietary CAR significantly reduced cecal *MYD88* and *NF-κB p65* expression (*P* < 0.05) and suppressed the LPS challenge-mediated increase in *IL-1β*, *IL-6*, *IL-8*, *TNF-α*, *TLR2*, *TLR4*, *MYD88*, *NF-κB*, *MAPK1*, *MAPK8*, and *MAPK14* expression levels (*P* < 0.05). These results indicated that dietary CAR ameliorated cecal inflammation in LPS-challenged rabbits.

### Intestinal morphology

**Figure 3:**
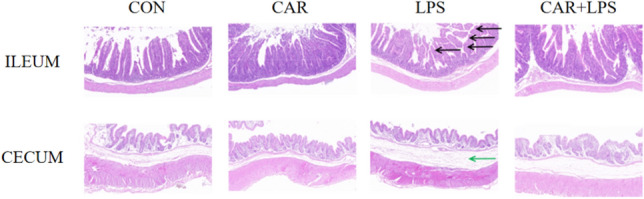
Effects of CAR on cecum histomorphology morphology of LPS-challenged rabbits. n = 4. There are significant the villi of the ileum injury at the black arrows, and the green arrow shows a significantly enlarged gap between the cecum mucosa and the sarcolemma layer.

We next assessed the effect of dietary CAR on intestinal morphology in response to LPS stimulation. As shown in Fig. 3, in the normal condition, ileal villi are closely connected. However, LPS stimulation significantly altered the morphology of the ileum. Compared with the controls, the villi were fractured and narrower, and the intestinal mucosa was significantly thinner. Under the influence of dietary CAR, LPS treatment did not result in any obvious injury to the rabbit ileum. Under normal conditions, the cecal mucus layer is closely connected to the sarcolemma. After LPS stimulation, the cecal sarcolemma was significantly damaged, and the space between the mucosal layer and the sarcolemma was enlarged, indicative of mucosa shedding. However, under the influence of dietary CAR, LPS did not cause obvious damage to the cecum of rabbits.

**Figure 4:**
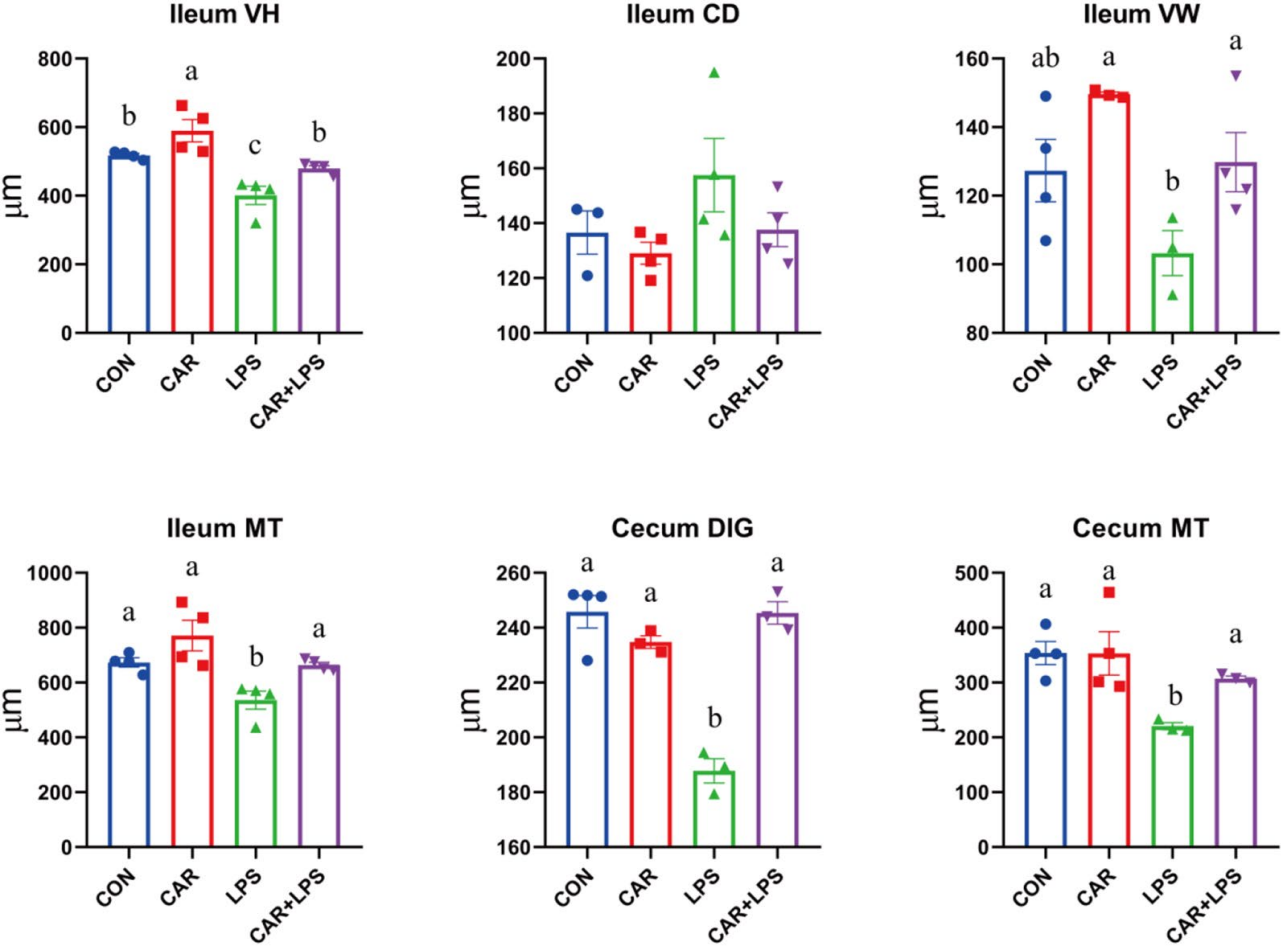
Effects of CAR on intestinal histomorphology morphology of LPS-challenged rabbits (μm). Values are the means of four replicates per treatment. VH: villus height; C: crypt depth; VW: villus width; MT: mucosal thickness; DIG: depth of intestinal gland.

The effects of the different treatments on intestinal morphology are presented in Fig. 4. LPS challenge led to a decrease in ileal villus height (VH), villus width (VW), and mucosal thickness (MT) (*P* < 0.05). Meanwhile, dietary CAR significantly increase ileal VH (*P* < 0.05) and mitigated the negative effects of LPS treatment on ileum VH, VW, and MT and cecal DIG and MT (*P* < 0.05). These results suggested that CAR supplementation promoted the recovery of intestinal morphology following LPS challenge.

### Expression of TJ genes in the cecum

**Figure 5:**
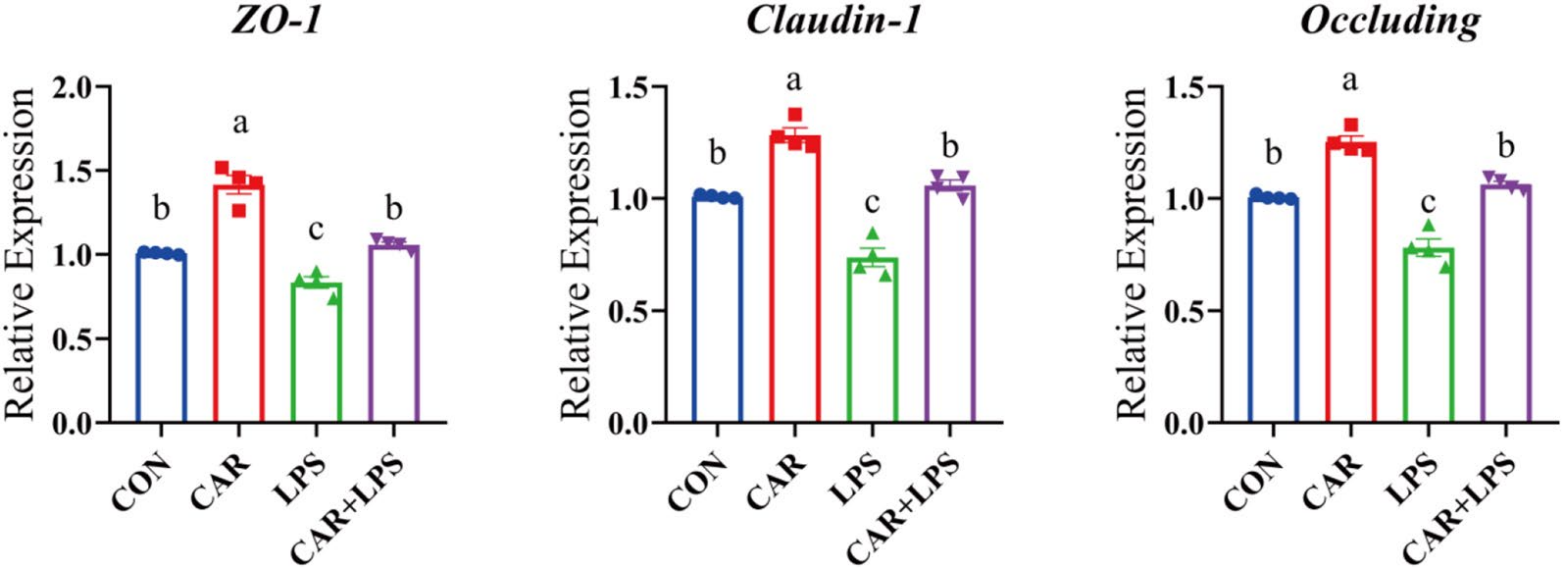
Effects of dietary CAR on cecum TJ genes of LPS-challenged rabbits. Values are the means of four replicates per treatment.

The effects of the different treatments on the expression of TJ genes in the cecum of rabbits are shown in Fig. 5. LPS challenge significantly reduced the cecal expression of the *ZO-1*, *claudin*-1, and *occludin* genes (*P* < 0.05), whereas the opposite was observed with dietary CAR (*P* < 0.05). Furthermore, the addition of CAR to the diet of rabbits blocked the negative effects of LPS challenge on the expression of the three genes (*P* < 0.05). These results suggested that CAR supplementation fostered the recovery of intestinal barrier function in rabbits following LPS insult.

### SCFAs in cecal digesta

**Figure 6:**
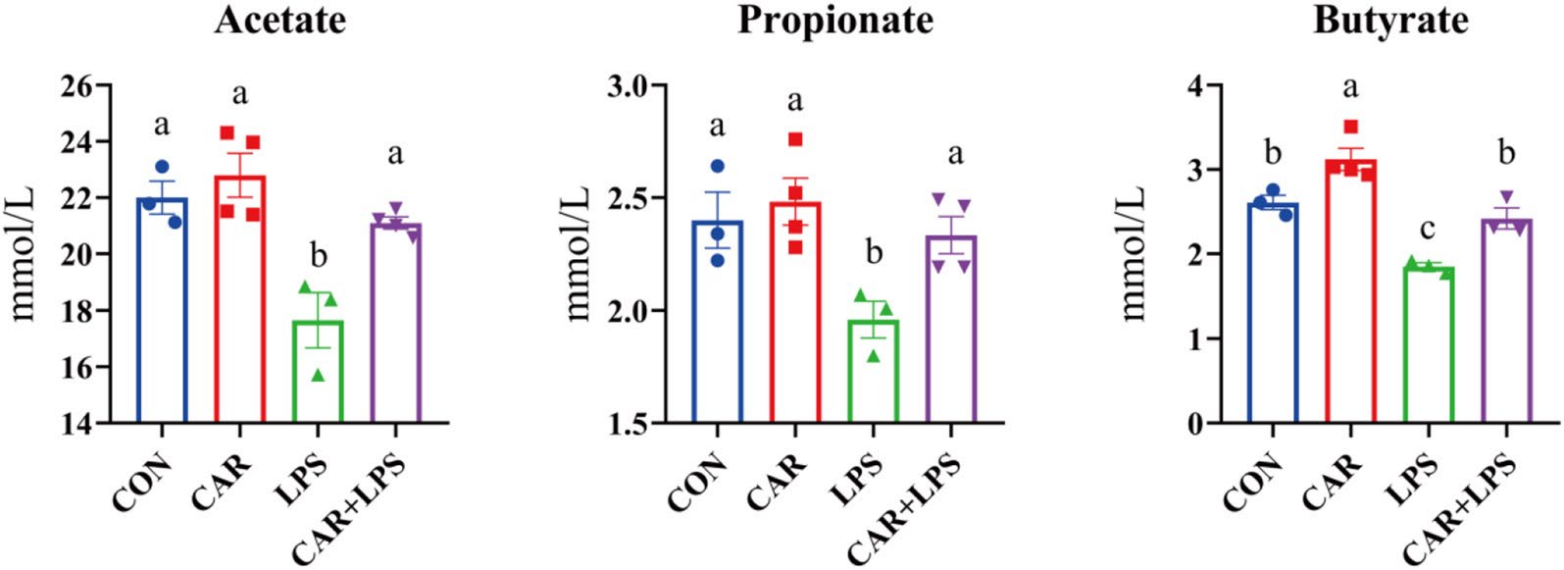
Effects of dietary CAR on SCFA in cecum of LPS-challenged rabbits (mmol/L). Values are the means of four replicates per treatment.

The effects of treatment on SCFAs contents are presented in Fig. 6. LPS challenge led to a decrease in acetate, propionate, and butyrate contents (*P* < 0.05). However, dietary CAR significantly increased butyrate levels (*P* < 0.05) and alleviated the LPS challenge-mediated reduction in the contents of acetate, propionate, and butyrate (*P* < 0.05). These results suggested that CAR supplementation reversed the negative effects of LPS on cecal SCFAs contents.

### Quantification of cecal bacteria

**Figure 7:**
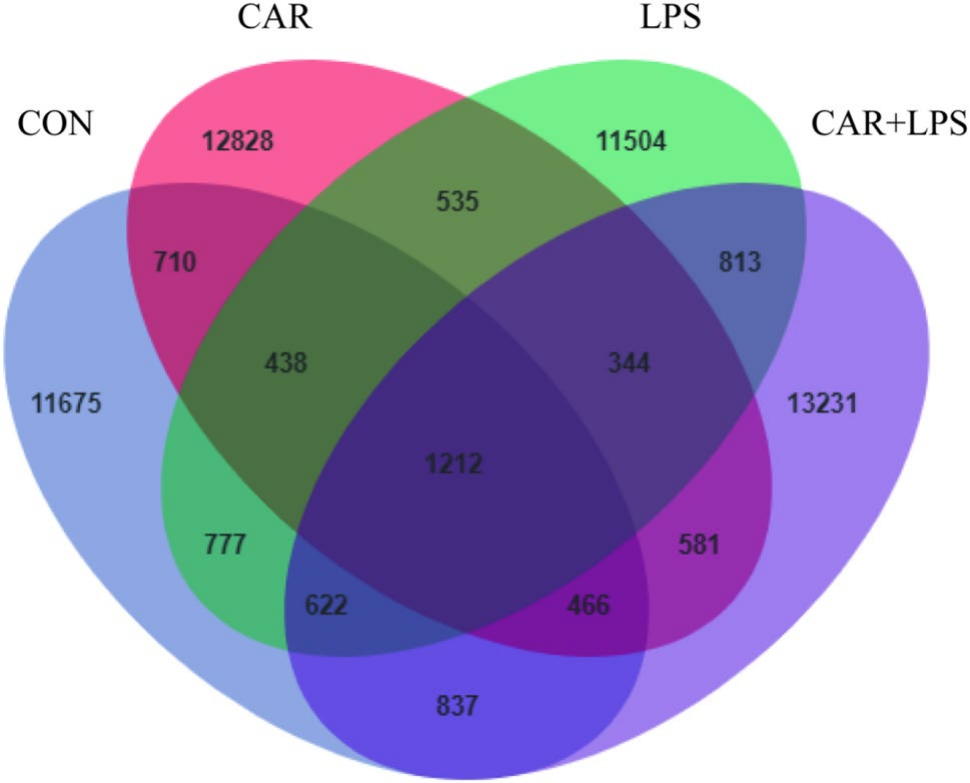
Effects of dietary CAR on cecum microorganisms Veen diagram of LPS-challenged rabbits. n = 4.

**Figure 8:**
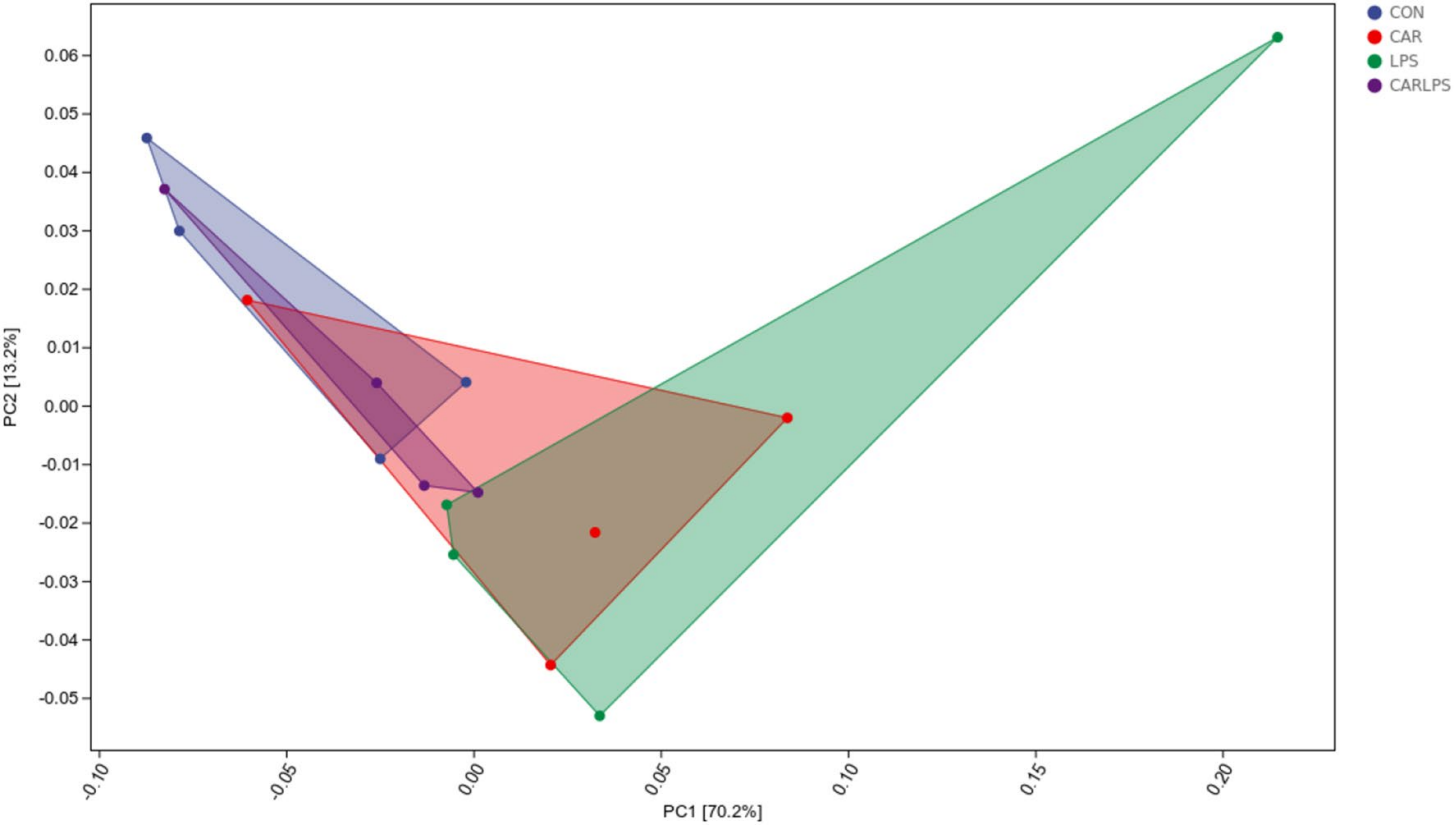
Effects of dietary CAR on cecum microorganisms PCA of LPS-challenged rabbits. n = 4.

**Figure 9:**
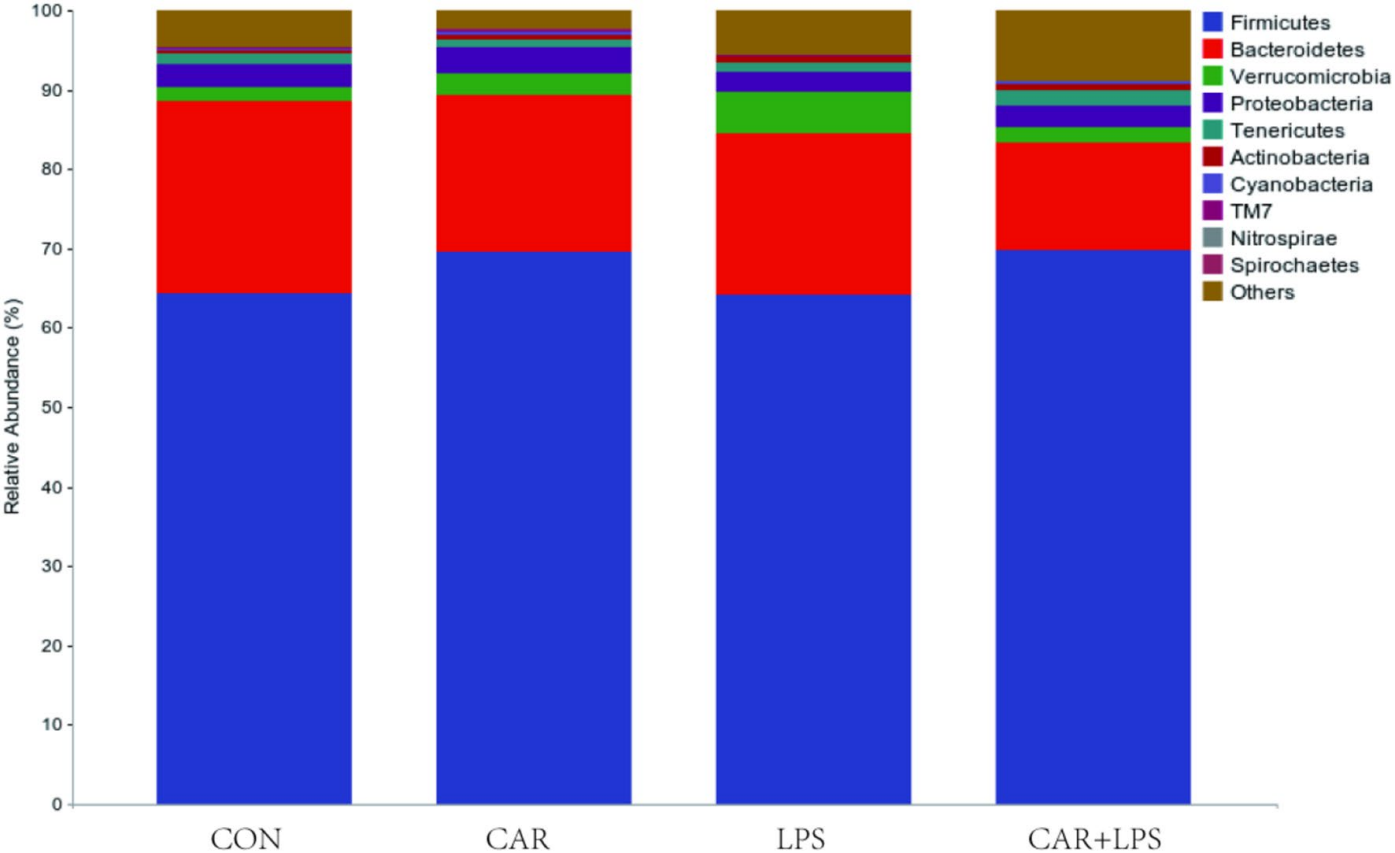
Effects of dietary CAR on cecum microorganisms phylum of LPS-challenged rabbits. n = 4.

**Figure 10:**
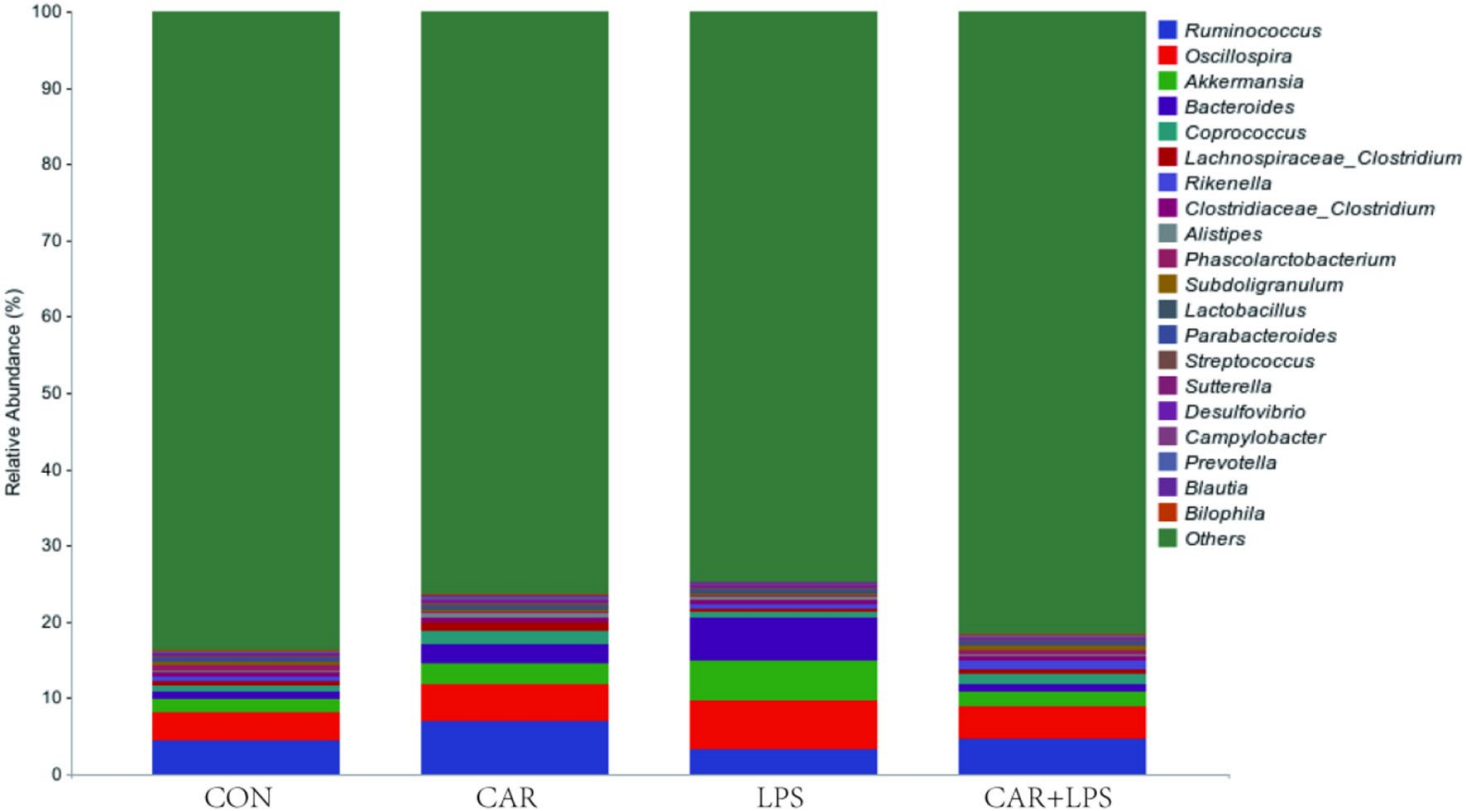
Effects of dietary CAR on cecum microorganisms genus of LPS-challenged rabbits. n = 4.

High-throughput sequencing of the 16S rRNA gene was performed to reveal the role of the cecal microbiota in the CAR-mediated recovery of intestinal injury induced by LPS challenge. As seen in the Venn diagram in Fig. 7 11,675, 12,828, 11,504, and 13,231 operational taxonomic units (OTUs) were identified in the CON, CAR, LPS, and CAR + LPS groups, respectively; 1212 OTUs were found to be shared among the four groups (Fig. 7). The microbial diversity within an individual sample was assessed by the Chao1, Faith’s PD, Shannon, and Simpson indices, but no alteration among all the groups was observed (Table 3). Next, PCA was performed on the OTUs to assess the similarities and differences between samples and groups (Fig. 8). The results revealed that the cecal microbiota in the LPS group was separated from that in the CON, CAR, and CAR + LPS groups. Taxonomic profiling indicated that Firmicutes accounted for most of the intestinal bacteria of rabbits (Fig. 9). No differences in relative bacterial abundance were observed among all the groups at the phylum level (Table 4). The relative abundance of the 20 dominant genera in each group at the genus level was also analyzed. *Ruminococcus* and *Oscillospira* were found to be the main genera in the four treatment groups (Fig. 10). As shown in Table 5, LPS challenge significantly reduced *Ruminococcus* abundance in the intestine of rabbits (*P* < 0.05). In contrast, CAR provision in the diet increased *Ruminococcus* abundance (*P* < 0.05) and alleviated the LPS stimulation-induced reduction in the relative abundance of members of this genus (*P* < 0.05). Combined, these results suggested that CAR supplementation mitigates the LPS stimulation-induced dysbiosis in the cecum of rabbits.

**Table 3:** Effect of dietary CAR on the Alpha diversity on cecum microbiota diversity of LPS-challenged rabbits %

Items	CON	CAR	LPS	CAR + LPS	*P*-value
Chao1	5182.93 ± 199.09	4979.52 ± 263.20	5041.33 ± 191.41	5439.94 ± 290.88	0.56
Faith-pd	361.26 ± 20.44	349.50 ± 19.42	330.37 ± 8.66	380.13 ± 23.47	0.34
Shannon	9.89 ± 0.28	9.93 ± 0.14	9.59 ± 0.17	10.31 ± 0.19	0.15
Simpson	0.99 ± 0.01	1.00 ± 0.00	0.99 ± 0.00	1.00 ± 0.00	0.33

Values are the means of four replicates per treatment.

**Table 4:** Effects of dietary CAR on cecum microorganisms phylum of LPS-challenged%

Items	CON	CAR	LPS	CAR + LPS	*P*-value
Firmicutes	64.27 ± 3.75	69.52 ± 4.63	64.10 ± 2.75	69.80 ± 2.55	0.51
Bacteroidetes	24.35 ± 4.36	19.83 ± 2.91	20.40 ± 4.82	13.47 ± 1.01	0.25
Verrucomicrobia	1.75 ± 0.65	2.65 ± 0.93	5.17 ± 1.93	2.07 ± 0.43	0.19
Proteobacteria	2.86 ± 0.60	3.32 ± 0.92	2.52 ± 0.25	2.63 ± 0.22	0.77
Tenericutes	1.25 ± 0.19	0.94 ± 0.34	1.27 ± 0.38	2.06 ± 0.41	0.16
Actinobacteria	0.55 ± 0.10	0.59 ± 0.08	0.67 ± 0.07	0.74 ± 0.08	0.40
Cyanobacteria	0.16 ± 0.01	0.53 ± 0.22	0.14 ± 0.02	0.24 ± 0.07	0.13
TM7	0.15 ± 0.09	0.14 ± 0.05	0.12 ± 0.04	0.13 ± 0.04	0.99
Nitrospirae	0.02 ± 0.01	0.03 ± 0.01	0.03 ± 0.01	0.03 ± 0.01	0.82
Spirochaetes	0.00 ± 0.00	0.04 ± 0.02	0.02 ± 0.02	0.01 ± 0.01	0.23

Values are the means of four replicates per treatment.

**Table 5:** Effects of dietary CAR on cecum microorganisms genus of LPS-challenged%

Items	CON	CAR	LPS	CAR + LPS	*P*-value
*Ruminococcus*	4.45 ± 0.27^b^	7.04 ± 0.36^a^	3.22 ± 0.20^c^	4.58 ± 0.42^b^	< 0.01
*Oscillospira*	3.57 ± 0.68	4.80 ± 1.52	6.49 ± 1.74	4.24 ± 0.58	0.42
*Akkermansia*	1.74 ± 0.64	2.64 ± 0.92	5.17 ± 1.94	2.07 ± 0.43	0.19
*Bacteroides*	1.09 ± 0.55	2.53 ± 1.61	5.63 ± 4.67	0.98 ± 0.24	0.54
*Coprococcus*	0.76 ± 0.14	1.68 ± 0.66	0.75 ± 0.04	1.30 ± 0.22	0.24
*Lachnospiraceae_Clostridium*	0.48 ± 0.08	1.22 ± 0.49	0.38 ± 0.12	0.55 ± 0.19	0.18
*Rikenella*	0.62 ± 0.33	0.08 ± 0.06	0.69 ± 0.68	1.12 ± 0.81	0.63
*Clostridiaceae_Clostridium*	0.59 ± 0.09	0.60 ± 0.13	0.49 ± 0.02	0.61 ± 0.12	0.83
*Alistipes*	0.32 ± 0.07	0.54 ± 0.19	0.38 ± 0.16	0.31 ± 0.05	0.60
*Phascolarctobacterium*	0.66 ± 0.36	0.10 ± 0.09	0.14 ± 0.14	0.58 ± 0.15	0.17
*Subdoligranulum*	0.38 ± 0.06	0.32 ± 0.09	0.23 ± 0.05	0.44 ± 0.13	0.40
*Lactobacillus*	0.31 ± 0.04	0.29 ± 0.06	0.37 ± 0.10	0.30 ± 0.06	0.83
*Parabacteroides*	0.36 ± 0.19	0.27 ± 0.17	0.29 ± 0.09	0.26 ± 0.09	0.96
*Streptococcus*	0.20 ± 0.04	0.30 ± 0.03	0.25 ± 0.05	0.20 ± 0.04	0.29
*Sutterella*	0.12 ± 0.03	0.34 ± 0.19	0.12 ± 0.03	0.15 ± 0.06	0.40
*Desulfovibrio*	0.15 ± 0.07	0.17 ± 0.14	0.24 ± 0.15	0.13 ± 0.11	0.93
*Campylobacter*	0.08 ± 0.02	0.14 ± 0.09	0.09 ± 0.04	0.22 ± 0.13	0.64
*Prevotella*	0.10 ± 0.02	0.14 ± 0.04	0.10 ± 0.01	0.16 ± 0.02	0.20
*Blautia*	0.11 ± 0.02	0.18 ± 0.09	0.13 ± 0.03	0.07 ± 0.02	0.43
*Bilophila*	0.11 ± 0.08	0.18 ± 0.03	0.15 ± 0.01	0.05 ± 0.01	0.08

Values are the means of four replicates per treatment.

### The correlations among inflammatory factors, TJ proteins, and gut microbiota

**Figure 11:**
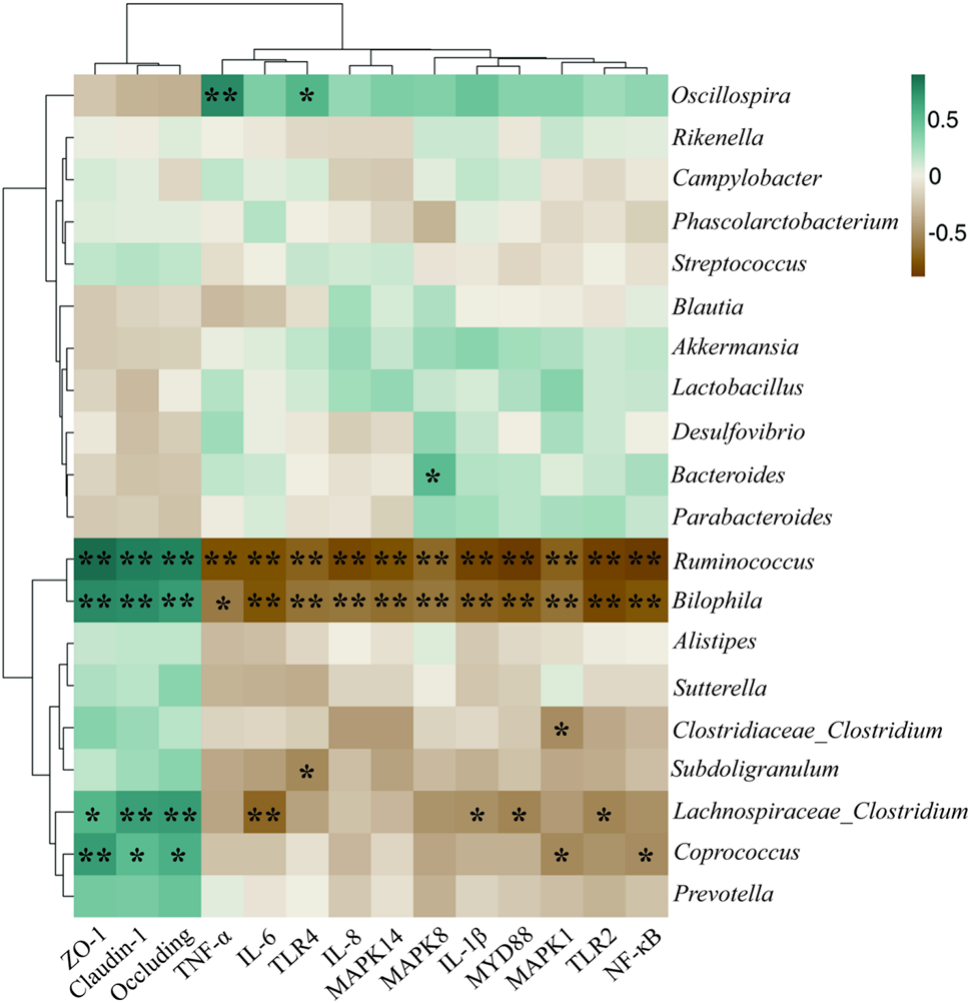
Heatmap of Spearman^’^s correlation between the gut microbiota and anti-inflammatory and TJ function. The intensity of the colors represented the degree of association (green, positive correlation; brown, negative correlation). Significant correlations were marked by *P < 0.05, **P < 0.01.

To further understand the role of the gut microbiota in the regulation of inflammation and TJ function, we performed a Spearman’s correlation analysis of the relationship among inflammatory factors, TJ proteins, and intestinal bacteria (Fig. 11). Eight microbial genera were found to be significantly correlated with inflammatory gene levels or the expression levels of genes coding TJ proteins. Among the genera upregulated by CAR, *Ruminococcus* was negatively correlated with the mRNA levels of *IL-1β*, *IL-6*, *IL-8*, *TNF-α*, *TLR2*, *TLR4*, *MYD88*, *NF-κB*, *MAPK1*, *MAPK8*, and *MAPK14* (*P* < 0.05) and positively correlated (*P* < 0.05) with those of *ZO-1*, *claudin 1*, and *occludin*.

## Discussion

The TLRs/MYD88/NF-κB pathway is the main pathway by which LPS induces inflammation in the intestine. TLRs are the major components of the innate immune system and their primary function is the recognition of intrinsic pathogen-associated molecules. LPS binds to and is recognized by TLRs, which is followed by the recruitment of the adaptor protein MYD88^28^. This leads to the activation of the NF-κB transcription factor, which stimulates the production and secretion of inflammatory factors such as TNF-α, IL-1β, IL-6, and IL-8^29^, and, in turn, leads to intestinal damage^30^. In the present study, the expression of the *TLR2* and *TLR4* genes was significantly increased in the rabbit intestine upon LPS stimulation; however, the addition of CAR to the diet mitigated this upregulation. Studies have found that TLR2 recognizes LPS through its lipid A part^31^. When the mouse *Tlr4* gene is mutated, it can no longer recognize LPS^32^, suggesting that CAR may reduce the ability of the intestine to recognize LPS by inhibiting the expression of *TLR2* and/or *TLR4*, thereby reducing the inflammatory response. MYD88 acts as a downstream factor of TLRs. After recognizing LPS, TLRs bind to the TIR domain of MYD88, leading to its activation and, subsequently, to that of NF-κB^29,33^. Activated NF-κB migrates to the nucleus, where it stimulates the production of inflammatory factors via its function as a transcription factor^34^. In our study, CAR suppressed the LPS-induced upregulation of *MYD88*, *NF-κB*, *TNF-α*, *IL-1β*, *IL-6*, and *IL-8*, indicating that CAR inhibited the LPS-induced activation of MYD88 and the NF-κB pathway. Dietary CAR also reduced the secretion of TNF-α, IL-1β, IL-6, and IL-8, thereby alleviating the intestinal inflammatory response. TNF-α can activate the *MAPK* genes and thus promote the further release of inflammatory factors, leading to an inflammatory cascade^35–37^. Several studies have shown that CAR can also inhibit the activation of *MAPK* genes by directly inhibiting the phosphorylation of MAPK1, MAPK8, and MAPK14^38–40^, which blocks the release of inflammatory factors. In the present study, we found that the MAPK-encoding genes were downregulated by CAR supplementation, likely due to the reduced expression of *TNFA*, which mitigated the amplification of the inflammatory response. In summary, the mechanism by which CAR alleviates LPS-induced intestinal inflammation in rabbits may involve the inhibition of the activation of TLRs/NF-κB/TNF-α/MAPK pathway-related genes, leading to a decrease in the production and secretion of TNF-α, IL-1β, IL-6, and IL-8.

Intestinal integrity is crucial for the development of animals and intestinal damage often leads to feeding intolerance and weakened nutrient absorption capacity^41^. VH, VW, crypt depth, and MT are important indicators of intestinal integrity. Intestinal villi are formed by intestinal stem cells that continuously self-renew and migrate upward. The intestinal mucosa, composed of intestinal epithelial cells^42^, is made up of ring-shaped folds comprising numerous villi. In the present study, we found that CAR significantly suppressed the reduction in VH, VW, and ileal and cecal mucosa thickness in rabbits after LPS stimulation. These results indicated that CAR could reverse the intestinal injury induced by LPS. The intestinal mucosal barrier prevents the translocation of bacteria and macromolecules, such as LPS, from the intestinal lumen to intestinal tissue^43^. The mechanical barrier function of the intestinal mucosa is mediated by proteins such as ZO-1, occludin, and claudin that form TJs between epithelial cells^44^. Occludin acts directly on the ZO-1 protein, which serves as a scaffold between the former (and claudin) and the cytoskeleton^45^; claudin, meanwhile, plays a key role in intestinal wall charge selectivity and the resistance of TJs^46^. Cell culture studies have shown that TNF-α can induce the reorganization of ZO-1, occludin, and claudin in cells. During this process, some genes occur protein loss and mislocalization, which suggests that TNF-α can disrupt TJs, thereby negatively affecting intestinal barrier function^47^. Studies have reported that ZO-1 mislocalization disrupts the stability of TJ complexes in both rats and piglets^48,49^. In the present study, adding CAR to the diet alleviated the marked increase in *TNF-α* expression and the significant reduction in *ZO-1*, *occludin*, and *claudin* 1 genes expression in the rabbit intestine after LPS stimulation. This result suggested that CAR mitigates the inhibitory effects of LPS on the expression of *ZO-1*, occludin, and claudin 1 by inhibiting that of *TNF-α*, thus reducing intestinal permeability. As the first line of intestinal defense, when the mechanical barrier is disrupted, LPS can enter the circulation through the mucosal barrier, leading to the release of inflammatory factors^50^. In addition, the upregulation of TLRs^51^, TNF-α^52^, IL-8^53^, NF-κB^54^, and MAPKs^55^ can promote apoptosis. Fukuda et al.^56^ showed that an increase in apoptosis reduced intestinal VH. In the present study, we found that CAR downregulated the LPS-induced increase in the expression of *TLR*s, *TNF-α*, *IL-8*, *NF-κB*, and *MAPK*s, suggesting that CAR can alleviate the intestinal injury induced by LPS by inhibiting the expression of these genes. The mechanism by which CAR alleviates LPS-induced intestinal injury in rabbits may involve the inhibition of the activation of TLRs/NF-κB/TNF-α/MAPK pathway-related genes. Increasing the expression of *ZO-1*, occludin, and claudin 1 and reducing the production of TLRs, TNF-α, IL-8, NF-κB, and MAPKs can alleviate the apoptosis of intestinal epithelial cells and intestinal stem cells, thereby reducing the destruction of the intestinal mucosa and villi and alleviating the LPS-induced intestinal damage. In this study, we demonstrated that CAR exerted a significant preventive effect on LPS-induced intestinal injury in rabbits. However, additional research is needed to further clarify this therapeutic effect of CAR.

A multitude of microorganisms colonize the gastrointestinal tract of animals, and these microorganisms interact with the intestinal epithelium, helping to maintain its integrity^57^. Intestinal microbes can benefit the host by regulating intestinal morphology^58^. In the present study, we found that LPS treatment modulated the structure of the cecal microbiota community in rabbits, which was similar to that previously reported^21^; however, this effect of LPS was alleviated by dietary CAR supplementation. Species-rich communities have been reported to enhance the stability of the intestinal microecology and reduce sensitivity to bacterial invasion and intestinal inflammation^59^. These results suggest that CAR may alleviate intestinal inflammation by regulating intestinal microbial community structure. In our study, CAR alleviated the LPS-induced reduction in the relative abundance of the genus *Ruminococcus* in the cecum of rabbits. Members of this genus are probiotics that promote gut health and play a crucial role in maintaining the metabolism of nutrients and microbial homeostasis^60^. In addition, *Ruminococcus* bacteria have been shown to produce SCFAs^61^, which are the main energy source of intestinal epithelial cells. SCFAs can promote the differentiation of intestinal epithelial cells as well as the development of intestinal villi, thereby maintaining intestinal morphology^62^. In addition, SCFAs provide energy for the differentiation of epithelial cells and consume 70% of intestinal oxygen^63,64^, thus generating a favorable environment for the growth and proliferation of *Ruminococcus* bacteria. The genus *Ruminococcus* was reported to be negatively correlated with inflammatory factors^61^. Sam et al.^65^ found that SCFAs can inhibit the secretion of inflammatory factors through the TLR pathway, which is consistent with the results of this study. Additionally, we found that *Ruminococcus* abundance and the relative mRNA expression levels of TJ proteins were positively correlated. Wan et al.^66^ demonstrated that SCFAs could significantly increase the relative expression of TJ protein-related genes by activating the AMPK signaling pathway, as well as inhibit the secretion of inflammatory factors, which also improved the relative expression levels of genes encoding TJ proteins. These results indicate that CAR can enhance the proliferation of *Ruminococcus* bacteria, which would be expected to lead to an increase in acetate, propionate, and butyrate contents in the cecum of rabbits. An increase in the levels of these SCFAs would subsequently result in a reduction of intestinal inflammation and the enhancement of the expression of intestinal TJ proteins in the cecum of rabbits, thereby alleviating the LPS-induced damage to intestinal morphology. These results provide a theoretical basis for understanding the relationship between CAR-mediated regulation of LPS-induced intestinal injury and the gut microbiota; however, further microbiome-based studies are needed to elucidate the mechanism underlying the anti-inflammatory effects of *Ruminococcus* bacteria.

## Conclusion

In summary, we found that dietary CAR supplementation can alleviate and reverse LPS-induced intestinal injury in rabbits by reducing the inflammatory response and increasing the relative abundance of *Ruminococcus* bacteria. In addition, our results confirmed that CAR exerted an anti-inflammatory effect in LPS-treated rabbits, which may be related to the inhibition of the expression of genes in the TLRs/NF-κB/TNF-α/MAPK pathway (Supplementary Information).

## Supplementary Information

Supplementary Information.

## Data Availability

The datasets generated and/or analysed during the current study are available in the CNCB repository, GSA ID: CRA010835.
